# Semi-supervised learning in prostate MRI tumor detection approaches fully supervised performance on external validation

**DOI:** 10.1007/s00330-026-12324-x

**Published:** 2026-01-28

**Authors:** Eduardo H. P. Pooch, Georgios Agrotis, Lishan Cai, Mark Emberton, Taimur T. Shah, Hashim U. Ahmed, Regina G. H. Beets-Tan, Sean Benson, Tomas Janssen, Ivo G. Schoots

**Affiliations:** 1 Department of Radiology, The Netherlands Cancer Institute, Amsterdam, The Netherlands; 2 GROW Research Institute for Oncology and Reproduction, Maastricht University, Maastricht, The Netherlands; 3 Division of Surgery and Interventional Science, University College London, London, UK; 4 Department of Surgery and Cancer, Imperial College London, London, UK; 5 Department of Cardiology, Amsterdam University Medical Centers, University of Amsterdam, Amsterdam, The Netherlands; 6 Department of Radiation Oncology, The Netherlands Cancer Institute, Amsterdam, The Netherlands; 7 Department of Radiology and Nuclear Medicine, Erasmus University Medical Center Rotterdam, Rotterdam, The Netherlands

**Keywords:** Prostate cancer, Magnetic resonance imaging, Artificial intelligence, Semi-supervised learning

## Abstract

**Objective:**

To evaluate the diagnostic performance of semi-supervised learning models for aggressive prostate cancer detection on MRI compared to fully supervised models trained with additional expert annotations.

**Materials and methods:**

We used 1500 MRI scans from the PI-CAI challenge training subset. Positive scans had 220 human and 205 AI-generated annotations. The mtU-Net (proposed teacher-student semi-supervised approach) was compared to supervised (trained using only 220 human annotations) and semi-supervised (trained on human and AI-generated annotations) nnU-Net. The 205 AI-annotated scans were manually annotated, and a fully supervised model was trained. External validation was performed on a newly annotated dataset from the PROMIS study (*n* = 574, 403 lesions) and the Prostate158 dataset (*n* = 158, 126 lesions). Patient-level performance was evaluated using the area under the curve (AUC) and lesion-level detection (overlap > 0.10) using average precision (AP), along with 95% confidence Intervals (in brackets), and the DeLong test to compare AUCs against the supervised and fully supervised models.

**Results:**

The fully supervised nnU-Net showed the highest performance on the internal PI-CAI test set (AUC = 0.89 [0.87–0.91], AP = 0.65 [0.60–0.70]) and external validation datasets PROMIS (AUC = 0.68 [0.64–0.72], AP = 0.24 [0.20–0.29]) and Prostate158 (AUC = 0.87 [0.82–0.92], AP = 0.64 [0.56–0.72]), significantly outperforming the supervised baseline (*p* < 0.0 5). The proposed semi-supervised mtU-Net demonstrated close external validation performance on PROMIS (AUC = 0.66 [0.62–0.71], AP = 0.20 [0.16–0.25]) and Prostate158 (AUC = 0.86 [0.81–0.92], AP = 0.58 [0.49–0.67]), significantly outperforming the supervised baseline on both datasets (*p* = 0.047 and *p* = 0.014, respectively), and showing no significant difference to the fully supervised model (*p* = 0.199 and *p* = 0.702, respectively).

**Conclusion:**

In prostate MRI tumor detection, fully supervised learning performed best. However, in external validation, the semi-supervised methods demonstrated performance that approached that of the fully supervised model, proving a valuable approach when expert annotations are limited.

**Key Points:**

***Question**** The need for extensive expert voxel-level annotations delays the development of AI-based prostate cancer diagnostic tools and their implementation in clinical practice*.

***Findings**** The combination of pseudo-labeling with consistency regularization achieved performance comparable to that of fully supervised methods, demonstrating that data diversity matches the impact of expert annotation volume*.

***Clinical relevance**** Semi-supervised learning reduces dependence on expert annotations while maintaining detection accuracy, enabling the development of scalable, automated diagnostic tools for prostate cancer amid growing clinical workflow demands*.

**Graphical Abstract:**

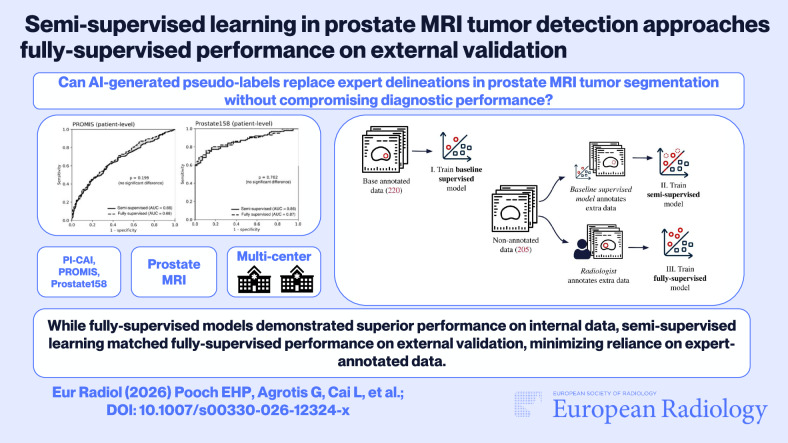

## Introduction

Prostate MRI is central to early cancer detection and treatment planning [1–3]. However, the reader-dependent nature of identifying aggressive cancer on MRI, defined as grade group (GG) ≥ 2, leads to significant variability across centers and an overall low positive predictive value [4]. Deep learning (DL) offers a way to standardize image interpretation and improve diagnostic performance [5], with the clinical goal of supporting lesion detection on MRI in men with suspected cancer to reduce unnecessary biopsies and missed cancers.

Developing robust lesion detection models is challenging because tumors occupy a small image fraction, have irregular shapes, and are often absent, making conventional 3D bounding‑box detection suboptimal due to substantial inclusion of normal tissue. Instead, voxel‑wise segmentation provides dense prediction maps that can be post‑processed into discrete lesion candidates and better capture irregular tumor morphology.

An obstacle in training DL-based medical image segmentation is the requirement for extensive voxel‑wise expert annotations. This process is time-consuming and costly. In the era of increasing demand for annotated data, semi-supervised learning (SSL) offers a solution by leveraging manually labeled and unlabeled data, reducing dependence on fully annotated datasets. A common SSL technique is pseudo-labeling [6], using predictions from a pre-trained supervised model to generate labels for unlabeled data, effectively expanding the dataset for further training.

Previous SSL studies in medical image segmentation have primarily focused on relatively simpler tasks, such as anatomical structure segmentation and tumor segmentation, when the target class is consistently present in all samples of the dataset [7–9]. These tasks can often achieve high performance with a few fully labeled samples (e.g., 20–50) with established segmentation methods like nnU-Net [10]. These SSL studies often simulate limited-label scenarios by using only a small subset of the available annotations in the dataset.

The PI-CAI challenge [11] is a machine learning competition to validate algorithms for detecting aggressive cancers on prostate bi-parametric (bp) MRI. It provided a platform for developing and comparing machine learning solutions, focusing on semi-supervised approaches, following the work of Bosma et al [12], which effectively implemented SSL by generating pseudo-annotations for prostate cancer segmentation only on pathologically confirmed scans. The top five approaches on the PI-CAI challenge [11] used a U-Net-based architecture [13] for segmentation and pseudo-labeling [12] as a semi-supervised technique, but primarily improved performance by adding other techniques, such as ensembling, incorporating zonal anatomy, self-supervised pre-training, or using clinical biomarkers like PSA density.

This study systematically investigates whether advancing the SSL mechanism can match the performance of adding more expert annotations for prostate cancer detection in a large-scale training and validation setup. To assess the model’s clinical utility, the study uses metrics for overall patient-level diagnostic performance and lesion-level detection. We hypothesize that SSL can achieve comparable performance to supervised models trained with fully annotated datasets. Furthermore, this study investigates the practical applicability of SSL to a real-world clinical challenge and its generalization to external datasets.

## Materials and methods

### Data

We used the data made available for the PI-CAI challenge [11], consisting of 1500 bi-parametric MRI (bpMRI) scans for training and two hidden test sets, Test 1 with 100 samples and Test 2 with 1000 hidden test samples, including 197 samples from one external center. The input data consists of three sequences: T2-weighted imaging (T2w), apparent diffusion coefficient (ADC) maps, and high *b*-value diffusion-weighted imaging (DWI). The training set included 425 histologically-confirmed GG ≥ 2 positive lesions, of which 220 were human-annotated for voxel-level lesions on bpMRI, while the remaining 205 samples were AI-annotated. The remaining 1075 cases were negative for GG ≥ 2 lesions, confirmed either by histopathology or a 3-year MRI follow-up. To evaluate the effect of increasing the number of human annotations, a prostate radiologist with 8 years of experience manually annotated the 205 AI-annotated images from the PI-CAI challenge dataset. These annotations were based on the bpMRI sequences available, and the radiologist was aware of the number of GG ≥ 2 lesions reported in histopathological findings. The annotations were made on ADC using the 3D Slicer software (version 5.0.3), and the original bpMRI sequences remained unaltered. The expert annotations performed for this study will be made publicly available.

One limitation of the PI-CAI [11] test sets is that most samples are obtained from the same centers as the PI-CAI training data. Therefore, we conduct external validation using two independent datasets, PROMIS [14] and Prostate158 [15], with a total of 732 scans to assess the generalizability of SSL-based segmentation models. Table 1 summarizes the characteristics of the three datasets.

**Table 1:** Patient characteristics and histopathological grade distribution for the three datasets

	PI-CAI (training)	PROMIS	Prostate158
Number of scans	1500	574	158
Age, years (± SD, range)	65.6 ± 7.2 (35–92)	63.5 ± 7.3 (< 50–83)	69.0 ± 9.0 (35–84)
PSA (IQR)	8.5 (7.1)	6.5 (3.8)	7.5 (6.3)
Histopathological confirmation (%)	1001/1500 (66.7)	574/574 (100)	158/158 (100)
*ISUP Grade Group*			
= 1	228	100	11
= 2	234	185	37
= 3	99	74	24
= 4	40	22	21
= 5	52	26	9
Number of lesions	450	396	126
Median lesion volume (IQR)	1077.36 (2106.61)	590.15 (800.17)	1000.19 (1885.75)

PROMIS is a multicenter confirmatory study from the UK. All 574 prostate cancer-suspected men in the study underwent 1.5 Tesla MRI and transperineal biopsy with a sampling interval of 5 mm. This saturation biopsy approach ensures a thorough examination of the prostate tissue, addressing the limitation of potential undersampling often associated with less rigorous biopsy techniques, such as targeted or systematic. Histopathological reports from the PROMIS trial guided the ground-truth lesion delineation, with 396 lesions delineated on ADC maps to create the ground-truth segmentations.

Prostate158 [15] is an open-source, single-center dataset containing 3 T bpMRI scans from 158 male patients with suspected prostate cancer from a German hospital. All patients underwent histopathological verification via biopsy or prostatectomy, with the cohort comprising 102 patients with verified prostate cancer (GG ≥ 1) and 56 control patients. All 102 positive cases contain expert annotations of observed PI-RADS ≥ 4 lesions.

### Evaluation metrics

Patient-level diagnostic performance was evaluated using the area under the receiver operating characteristic curve (AUC). For this computation, each patient scan was assigned the highest voxel prediction score as its likelihood of containing aggressive (GG ≥ 2) cancer. The AUC was then derived from the ROC curve, summarizing the model’s ability to discriminate between positive and negative cases across all thresholds.

Lesion-level detection performance was assessed using average precision (AP), representing the area under the precision-recall curve. Following the PI-CAI official evaluation pipeline (version 1.4.13), lesion candidates were extracted from the model’s softmax output maps using a per-case dynamic threshold based on the maximum score, followed by 3D connected component analysis. Components below a minimum voxel size are discarded, and the confidence score for each lesion candidate is set as the maximum value within that component. Predicted lesions were extracted and classified as a true positive if the intersection over union with a ground-truth lesion annotation was greater than 0.10. Predictions not meeting this criterion were classified as false positives (FP). AP was then calculated based on the precision and recall values derived from ranking all predicted lesion detections

The metric for overall performance was the PI-CAI challenge score, defined as the average of the patient-level AUC and the lesion-level AP. For statistical analysis, 95% confidence intervals (CIs) for both AUC and AP were determined using bootstrapping with 10,000 iterations. The significance of differences in patient-level AUC was evaluated using the DeLong test. For differences in lesion-level AP, a bootstrap test was employed. P-values from all comparisons were subsequently adjusted using the Holm-Bonferroni correction to control the false discovery rate across multiple tests. An adjusted *p*-value less than 0.05 was considered significant. All performance metrics are reported in the text as (AUC value [95% CI]; AP value [95% CI]; *p*-value, where applicable).

### Supervision strategies

**Fig. 1:**
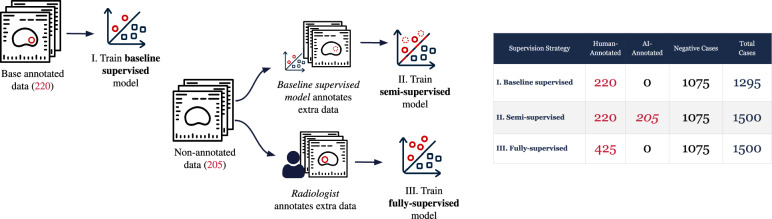
Experimental setup of the three different training strategies: baseline supervised (I), semi-supervised (II), and fully supervised (III) and the number of human-annotated and AI-annotated samples in each training strategy

To systematically evaluate the impact of different training data compositions, three distinct supervision strategies were established (Fig. 1). The training cohort for all strategies included 1045 cases that were negative for GG ≥ 2 cancer. For these negative scans, the entire image volume served as a “background” class, providing no lesion annotations and thereby training the models to recognize cancer-free features. The strategies differed in their use of the 425 positive cases. A baseline supervised learning model was trained using the 220 human-annotated samples. This supervised model was used to annotate samples identified as GG ≥ 2 positive based on the information on the reports, generating 205 AI-labeled samples [12], which were included in the PI-CAI challenge training set. These AI-labeled samples were used for the semi-supervised approaches. A second supervised model (referred to as fully supervised) was trained on newly annotated data by a radiologist on these 205 samples.

### Model architecture

The performances of all three strategies were evaluated using three different network architectures. Two well-established frameworks, the nnU-Net [10] and nnDetection [16], and, in addition, we propose a new architecture, mtU-Net, as a third semi-supervised approach.

The nnU-Net [10] is a state-of-the-art DL framework specifically designed for medical image segmentation tasks. It is based on the U-Net [13] approach for image segmentation and automatically configures data augmentation and architecture based on the specific dataset, often achieving top performance across different benchmarks. The nnDetection [16] is another framework built for medical image analysis, inspired by object detection architectures. It is built upon the Retina U-Net [17] architecture, which is a fusion of the Retina Net one-stage object detection approach [18] with the U-Net segmentation architecture [13]. It specializes in detecting objects by using anchor boxes, which are predefined bounding boxes of different sizes and aspect ratios. Concurrently, the network predicts the probability of object inclusion for each anchor box and performs pixel-level segmentation.

### mtU-Net

**Fig. 2:**
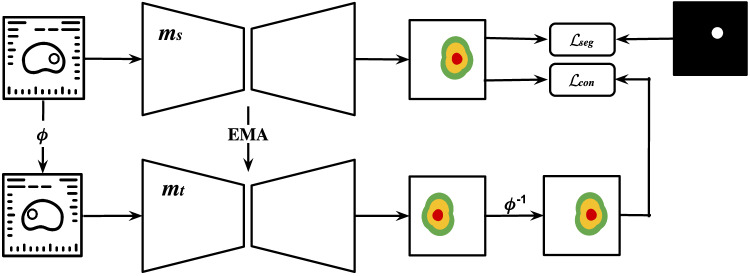
Architecture of mtU-Net. Each sample in the dataset is fed to a student model ${m}_{s}$ that outputs segmentation scores, and the segmentation loss ${{{{\mathscr{L}}}}}_{{seg}}$ is computed based on the predictions and ground-truth/pseudo-annotations. The same image receives a transformation $\phi$ and is fed to a teacher model ${m}_{t}$, which also generates patch scores. The output of the teacher model receives an inverse transformation ${\phi }^{-1}$, so the scores match the original image, and the difference between both outputs is added to the training loss as the ${{{{\mathscr{L}}}}}_{{con}}$ to ensure consistency. The student’s weights are updated with backpropagation, and the teacher’s weights are an exponential moving average (EMA) of the student’s weights

To extend the use of the non-annotated data, we propose mtU-Net, a 3D semi-supervised segmentation model that employs a teacher-student framework integrating pseudo-labeling and consistency regularization (Fig. 2). We call our method mtU-Net, based on the Mean-Teacher framework [19]. Mean-teacher approaches for semi-supervised segmentation have been widely explored in medical imaging segmentation [20–26]. In most existing methods, every image in the dataset contains the target class, so the teacher is continually anchored to the presence of voxel‑wise masks. When this assumption does not hold, as in diagnostic prostate MRI, where only a subset of scans are lesion-positive and annotated, a naïve mean-teacher setup can become unstable.

In this work, the proposed mtU‑Net explicitly adapts the mean‑teacher idea to this partially labeled, lesion‑detection setting by (i) restricting consistency to reversible spatial transformations and applying the inverse transform to the teacher output before computing the voxel‑wise consistency loss, ensuring anatomically aligned supervision [27], (ii) integrating pseudo‑labeling so that pathologically positive scans receive AI‑generated voxel‑wise tumor masks while negative scans are treated as background, allowing the model to learn negative cases, and (iii) a focal loss component in the supervised term to up‑weight rare lesion voxels relative to the abundant background.

## Results

### Internal model performance

Three DL model architectures were investigated: nnU-Net, nnDetection, and mtU-Net. For the nnU-Net and nnDetection architectures, models were trained and compared utilizing supervised, semi-supervised, and fully supervised strategies. The mtU-Net architecture was evaluated using a semi-supervised training strategy. Internal results are reported in Table 2.

**Table 2:** Performance metrics of each training strategy across the PI-CAI test sets. Area under the curve (AUC), average precision (AP), the average of these two metrics (Avg), and 95% confidence intervals are reported

		PI-CAI Validation (*n* = 100)				PI-CAI Test (*n* = 1000)	
Architecture	Strategy	AUC	AP	Avg	AUC	AP	Avg
nnU-Net	Supervised	0.74 [0.63–0.83]	0.46 [0.32–0.62]	0.60 [0.48–0.72]	0.80 [0.77–0.83]	0.45 [0.39–0.51]	0.63 [0.59–0.67]
	Semi-supervised	0.82 [0.73–0.90]	0.61 [0.46–0.77]	0.71 [0.61–0.83]	0.87 [0.84–0.89]	0.58 [0.52–0.63]	0.72 [0.69–0.76]
	Fully supervised	**0.89 [0.82–0.94]**	**0.73 [0.60–0.84]**	**0.81 [0.72–0.89]**	**0.89 [0.87–0.91]**	**0.65 [0.60–0.70]**	**0.77 [0.73–0.80]**
nnDetection	Supervised	0.74 [0.63–0.83]	0.27 [0.16–0.43]	0.50 [0.41–0.62]	0.79 [0.75–0.82]	0.39 [0.33–0.44]	0.59 [0.55–0.63]
	Semi-supervised	0.89 [0.81–0.95]	0.58 [0.43–0.74]	0.73 [0.64–0.83]	0.87 [0.85–0.90]	0.55 [0.49–0.60]	0.71 [0.68–0.75]
	Fully supervised	0.80 [0.70–0.88]	0.57 [0.43–0.71]	0.69 [0.57–0.79]	0.83 [0.80–0.85]	0.48 [0.43–0.54]	0.65 [0.62–0.69]
mtU-Net	Semi-supervised	0.85 [0.77–0.92]	0.68 [0.55–0.81]	0.77 [0.67–0.86]	0.86 [0.84–0.89]	0.58 [0.52–0.63]	0.72 [0.68–0.75]

The best results are in bold, and the second best are underlined

On the PI-CAI validation set (*n* = 100), the nnU-Net architecture trained with a fully supervised strategy achieved the highest performance (AUC 0.89 [0.82–0.94]; AP 0.73 [0.60–0.84]). The semi-supervised strategy showed the second-highest performance for the nnU-Net, followed by the nnU-Net baseline supervised strategy. For the nnDetection architecture, the semi-supervised strategy showed the highest results (AUC 0.89 [0.81–0.95]; AP 0.58 [0.43–0.74]), followed by the fully supervised and semi-supervised strategy, which presented the lowest AP over all models in the dataset. The mtU-Net semi-supervised strategy showed higher AUC and AP (AUC 0.85 [0.77–0.92]; AP 0.68 [0.55–0.81]) than the semi-supervised nnU-Net (AUC 0.82 [0.73–0.90]; AP 0.61 [0.46–0.77]), on this dataset.

On the PI-CAI test set (*n* = 1000), the nnU-Net architecture trained with a fully supervised strategy also achieved the highest performance (AUC 0.89 [0.87–0.91]; AP 0.65 [0.60–0.70]). The semi-supervised strategy was the second-highest performance of the nnU-Net architecture, followed by the supervised nnU-Net. For the nnDetection architecture, the semi-supervised strategy showed the highest results (AUC 0.87 [0.85–0.90]; AP 0.55 [0.49–0.60]), followed by the fully supervised and semi-supervised, which had the lowest overall performance. In contrast to the validation set, in the PI-CAI test set, the mtU-Net semi-supervised strategy (AUC 0.86 [0.84–0.89]; AP 0.58 [0.52–0.63]) shows numerically close performance metrics to the nnU-net semi-supervised strategy (AUC 0.87 [0.84–0.89]; AP 0.58 [0.52–0.63]).

### External model performance

**Fig. 3:**
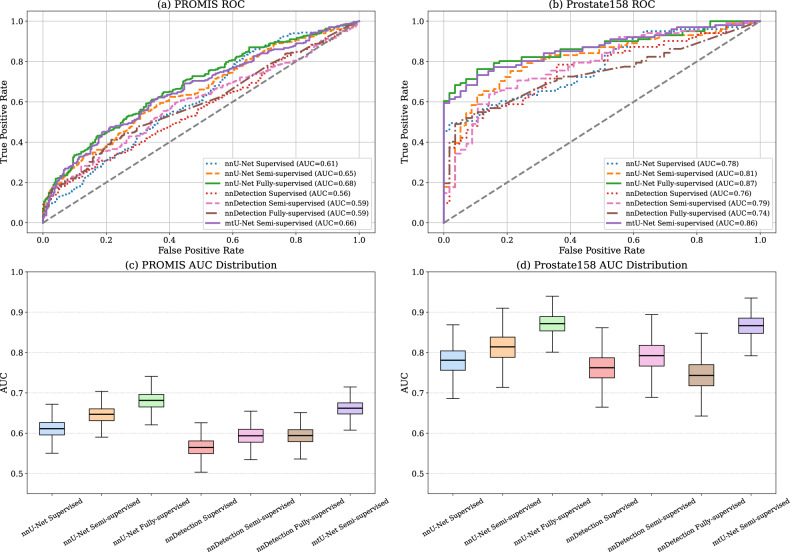
Receiver operating characteristic (ROC) curves of all models across the external validation datasets. **a** PROMIS and **b** Prostate158. Area under the curve (AUC) distribution box plots. **c** PROMIS and **d** Prostate158

**Fig. 4:**
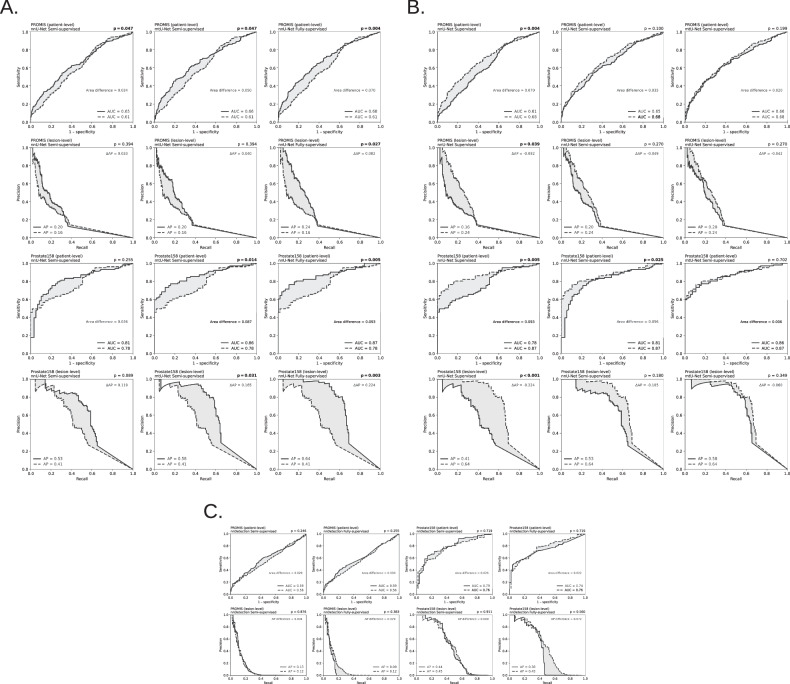
Statistical analysis of model performance on the two external datasets (PROMIS and Prostate158). The analysis consists of pairwise comparisons against three distinct baselines. **A** Comparison of model performance against the nnU-Net Supervised baseline. **B** Comparison of model performance against the nnU-Net Fully supervised baseline. **C** Comparison of the nnDetection Semi-supervised and Fully supervised models against the nnDetection Supervised baseline. For each comparison, the top row displays patient-level receiver operating characteristic (ROC) curves, with area under the curve (AUC) values and *p*-values from the DeLong test. The bottom row displays lesion-level Precision-Recall (PR) curves, with average precision (AP) values and *p*-values from the bootstrap test. The shaded area in each plot visualizes the difference between the model (solid line) and the baseline (dashed line). All *p*-values were corrected using the Holm-Bonferroni method to control the false discovery rate across multiple tests

An overview of external performance on PROMIS (*n* = 574) and Prostate158 (*n *= 158) is shown in Table 3 and Fig. 3. On external validation, the nnU-Net Fully supervised strategy demonstrated statistically significantly higher (*p* < 0.05) metrics over the nnU-Net Supervised baseline (Fig. 4A) on PROMIS for both patient-level AUC (0.68 [0.64–0.72], *p* = 0.004) and lesion-level AP (0.24 [0.19–0.29], *p* = 0.027). Results on Prostate158 also show statistically significantly higher values in AUC (0.87 [0.82–0.92], *p* = 0.005) and AP (0.64 [0.56–0.72], *p* = 0.003). When comparing the segmentation-based semi-supervised strategies, on the nnU-Net Semi-supervised, a statistically significant difference was limited to the AUC on the PROMIS dataset (0.65 [0.60–0.69], *p* = 0.047), whereas the mtU-Net Semi-supervised strategy showed statistically significant differences over the supervised baseline on the Prostate158 dataset for both AUC (0.86 [0.81–0.92], *p* = 0.014) and AP (0.58 [0.49–0.67], *p* = 0.031). On the PROMIS dataset, a statistically significant difference was observed for AUC (0.66 [0.62–0.71], *p* = 0.047), but not for AP (0.20 [0.16–0.25], *p* > 0.39). When comparing the semi-supervised methods with the nnU-Net fully supervised (Fig. 4B), mtU-Net showed no statistically significant difference in AUC on either the PROMIS (0.66 [0.62–0.71], *p* = 0.199) or Prostate158 (0.86 [0.81–0.92], *p* = 0.702) dataset, while the nnU-Net Semi-supervised model showed a non-significant difference in performance on PROMIS only (0.65 [0.60–0.69], *p* = 0.100). For the nnDetection-based models (Fig. 4C), no statistically significant differences were observed when comparing the semi-supervised and fully supervised models to the supervised baseline.

**Table 3:** Area under the curve (AUC), average precision (AP), the average of these two metrics (Avg), and 95% confidence intervals for different models across PROMIS and Prostate158 datasets

		PROMIS (*n* = 574)			Prostate158 (*n* = 158)		
Architecture	Strategy	AUC	AP	Avg	AUC	AP	Avg
nnU-Net	Supervised	0.61 [0.57–0.66]	0.16 [0.12–0.20]	0.39 [0.36–0.42]	0.78 [0.71–0.84]	0.41 [0.32–0.51]	0.60 [0.54–0.66]
	Semi-supervised	0.65 [0.60–0.69]	0.20 [0.16–0.24]	0.42 [0.39–0.45]	0.81 [0.74–0.88]	0.53 [0.44–0.62]	0.67 [0.61–0.73]
	Fully supervised	**0.68 [0.64–0.72]**	**0.24 [0.20–0.29]**	**0.46 [0.43–0.49]**	**0.87 [0.82–0.92]**	**0.64 [0.56–0.72]**	**0.75 [0.70–0.80]**
nnDetection	Supervised	0.56 [0.52–0.61]	0.12 [0.09–0.16]	0.34 [0.31–0.37]	0.76 [0.68–0.83]	0.45 [0.36–0.54]	0.61 [0.55–0.67]
	Semi-supervised	0.59 [0.55–0.64]	0.13 [0.09–0.16]	0.36 [0.33–0.39]	0.79 [0.72–0.86]	0.44 [0.35–0.53]	0.62 [0.56–0.68]
	Fully supervised	0.60 [0.55–0.64]	0.10 [0.06–0.13]	0.34 [0.32–0.37]	0.74 [0.67–0.81]	0.38 [0.29–0.47]	0.56 [0.50–0.62]
mtU-Net	Semi-supervised	0.66 [0.62–0.71]	0.20 [0.16–0.25]	0.43 [0.40–0.46]	0.86 [0.81–0.92]	0.58 [0.49–0.67]	0.72 [0.67–0.77]

The best results are in bold, and the second best are underlined

If we analyze the model performance at a fixed operating point selected to achieve a false-positive rate (FPR) of 0.50 on the combined external validation data (PROMIS and Prostate158, *n* = 732), we can compare model performance, measured by the absolute number of missed cancers. At a 0.50 FPR threshold, the nnU-Net supervised baseline would miss cancer in a total of 145 patients, while the fully supervised model would miss cancer in only 95 patients. Both semi-supervised approaches significantly improved upon the baseline. At this threshold, the nnU-Net Semi-supervised model would miss cancer in 119 patients, whereas our proposed mtU-Net model would miss it in 102 patients.

## Discussion

This study systematically evaluated semi‑supervised learning for prostate cancer detection on MRI to assess whether advanced SSL strategies can substitute for additional expert annotations in a large‑scale, clinically realistic setting. While fully supervised nnU‑Net achieved the highest internal performance, semi‑supervised approaches, particularly the proposed mtU‑Net, closely approached the fully supervised benchmark in external data, aligning with the findings of previous studies [12, 28]. These findings indicate that, beyond a certain point, increasing the volume of internal human annotations yields limited gains on external cohorts, whereas leveraging unlabeled data via SSL can improve robustness and generalizability.

By integrating consistency regularization with pseudo-labeling, our proposed mtU-Net architecture was specifically designed to mitigate the impact of noise from AI-generated annotations, thereby improving model generalizability. This was evidenced by its consistently strong performance across the internal PI-CAI test sets and external PROMIS and Prostate158 datasets, as well as being the only semi-supervised approach that showed statistically significant improvement over the supervised baseline on Prostate158 and closely aligned ROC curves with the fully supervised model.

**Fig. 5:**
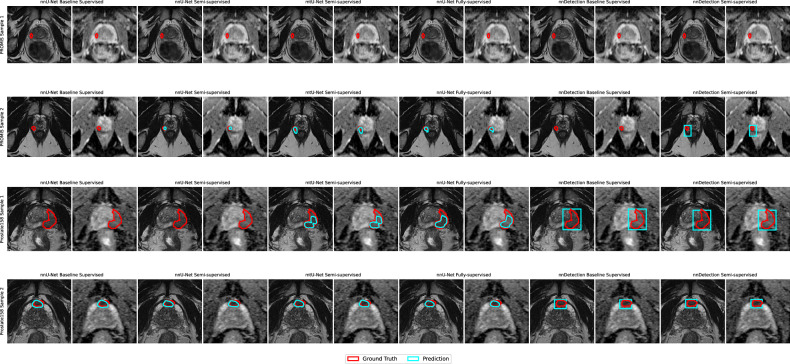
Examples of true positive cases and failure cases for 2 samples in the PROMIS and the Prostate158 dataset. Images show prediction and ground truth for each model over the T2-weighted sequences and apparent diffusion coefficient (ADC) maps

Despite strong patient-level performance, lesion-level gaps remain. On PROMIS, neither semi-supervised strategy improved lesion-level AP over the supervised baseline (*p* > 0.39), indicating that SSL still falls short of fully supervised models for precise lesion localization, particularly on smaller lesions (Fig. 5). A likely explanation is spatial noise in pseudo-labels, which provide a useful signal for patient-level classification but can mislocalize tumors, encouraging spatially inaccurate voxel-wise predictions.

The marked performance drop on PROMIS compared to PI-CAI and Prostate158 likely reflects both stricter ground truth and domain shift. PROMIS used saturation biopsy for all 574 men, minimizing verification bias relative to PI-CAI and Prostate158, where clinically driven reference standards can underrepresent non-MR-visible cancers. PROMIS also consists entirely of older 1.5 T scans collected in 2012, whereas PI-CAI and Prostate158 predominantly include more recent 3 T data, creating a substantial technical mismatch. A similar degradation was reported by Hering et al [29], whose system’s AUC decreased from 0.91 on the PI-CAI cohort to 0.79 on the PROMIS cohort.

The effectiveness of this approach underscores that architectural suitability varies; not all architectures handle different learning strategies or potentially inaccurate AI-generated annotations equally well. For example, nnDetection demonstrated this variability, with metrics indicating comparative underperformance in fully supervised settings relative to semi-supervised applications, suggesting that a lesser dependency on high-quality annotations might benefit certain model types.

This study has potential limitations. The 205 manual annotations used for the fully supervised training were delineated by one radiologist without access to full histopathology reports, and inter-reader variability was not assessed. For the PROMIS dataset, although the ground truth was guided by saturation biopsy reports and all patients underwent biopsy, an expert radiologist had to correlate these histopathological maps with mpMRI regions, a process that could introduce variation. For the Prostate158 dataset, the lesion-positive subset also contains GG = 1 cases (11 samples). The external datasets can contain samples with different acquisition protocols, vendors and field strengths than the data on the training set. The AI-generated annotations were based on a specific methodology [12], and the effectiveness of SSL might differ depending on the quality and characteristics of pseudo-annotations generated by other techniques. Location-based report-guided SSL methods [30] can further denoise pseudo-annotations by extracting lesion location information from free-text radiology reports to refine AI-generated labels. However, they depend on access to full reports, which is generally not available for public prostate MRI datasets.

In conclusion, adding expert annotations improved internal performance but offered limited gains in generalizability compared to SSL. Semi-supervised models achieved similar external validation performance to fully supervised learning by using non-annotated data, highlighting that data volume and diversity, not just expert annotations, drive robustness. This supports SSL as a valuable and viable approach for aggressive prostate tumor detection, maintaining competitive, generalizable performance while reducing the need for extensive, costly expert annotations.

## Supplementary information

ELECTRONIC SUPPLEMENTARY MATERIAL

## Abbreviations

ADC: Apparent diffusion coefficient
AP: Average precision
AUC: Area under the receiver operating characteristic curve
bpMRI: Bi-parametric MRI
CI: Confidence interval
DL: Deep learning
FP: False positives
GG: Grade group
SSL: Semi-supervised learning
T2w: T2-weighted imaging
