# Supplementary material for: Semi-supervised learning in prostate MRI tumor detection approaches fully supervised performance on external validation
Source: Eur Radiol. 2026 Jan 28;36(6):5011–21. doi: 10.1007/s00330-026-12324-x (PMC13212706; doi:10.1007/s00330-026-12324-x)
Supplement: Supplementary file 1 — ELECTRONIC SUPPLEMENTARY MATERIAL [file 330_2026_12324_MOESM1_ESM.pdf]

Semi-Supervised Learning in Prostate MRI Tumor Detection Approaches Fully-Supervised Performance on External Validation

ELECTRONIC SUPPLEMENTARY MATERIAL

1. Supplementary Material

1.1. PROMIS annotation

PROMIS is a multicenter confirmatory study from the UK. All 574 prostate cancer-suspected men in the study underwent 1.5 Tesla MRI and transperineal biopsy with a sampling interval of 5mm. This saturation biopsy approach ensures a thorough examination of the prostate tissue, addressing the limitation of potential undersampling often associated with less rigorous biopsy techniques, such as targeted or systematic. Histopathological reports from the PROMIS trial guided the ground truth lesion delineation. The reports were organized in two formats: either on a per-core basis (92 patients) or grouped by 20 Barzell zones (482 patients). Transperineal maps derived from these reports indicated the precise location and Gleason grade of identified GG≥2 tumors. An expert radiologist correlated these histopathological maps with corresponding regions on mpMRI. To identify suspicious lesions on the images, the radiologist applied PI-RADS v2.1 criteria, such as low T2W signal and restricted diffusion (e.g., low signal on ADC maps). Following identification, the 396 lesions were delineated on ADC maps to create the ground-truth segmentations. Multifocality was addressed by delineating multiple lesions whenever the transperineal map revealed non-adjacent malignant findings. The reader was blinded to clinical variables not present in the histopathology reports, such as patient age and PSA levels.

1.2. mtU-Net architecture

The training strategy consists of using two models with identical architecture, which are denoted student m_s and teacher m_t. At every training iteration, both models are fed the same input x with a different transformation, and then a consistency loss is computed based on the distance between both models' predictions.

The student weights Θ^s are updated via loss optimization, and the teacher weights Θ^t are updated via an exponential moving average (EMA) of the student weights after each training step e. A hyperparameter ρ controls the EMA decay rate to update the teacher's weights, as in $\Theta_e^t = \rho\Theta_{e-1}^t + (1-\rho)\Theta_e^s$.

Consistent with the PI-CAI baseline approach [6], the segmentation loss $\mathcal{L}_{seg}$ is a combination of focal loss $\mathcal{L}_{fl}$ [18] with cross-entropy loss $\mathcal{L}_{ce}$ between the student model's predictions $m_s(x)$ and the ground-truth (either human or AI annotations). The focal loss $\mathcal{L}_{fl} = -\alpha\big(1 - m_s(x)\big)^\gamma \log\big(m_s(x)\big)$ ensures a larger weight for the positive class and the less confident predictions by using weighting factor $\alpha = 0.75$ and the focusing parameter $\gamma = 2$, which is advantageous for segmenting smaller structures such as tumors. Here, α balances the importance of positive and negative examples, while γ focuses the loss on less-confident examples, thereby improving the model's ability to accurately segment small lesions. The weight of the focal loss is controlled by a hyperparameter which we choose as $\lambda_{fl} = 0.5$.

$$\mathcal{L}_{seg} = \lambda_{fl}\mathcal{L}_{fl} + \big(1 - \lambda_{fl}\big)\mathcal{L}_{ce} \qquad (1)$$

To enforce consistency regularization, we introduce a new loss term to the optimization function. In a classification scenario proposed by [19], the labels remain the same if the input is rotated or shifted; however, in a segmentation task, these functions affect the output, which is a problem when computing the consistency metric. Based on consistency regularization for object detection [20], we propose a straightforward and reversible augmentation strategy denoted as $\phi(\cdot)$, which flips the input image x horizontally and, consequently, causes the segmentation output of the model to be flipped. As shown in Equation 2, to compute the consistency loss $\mathcal{L}_{con}$, we also apply the transformation $\phi^{-1}(\cdot)$ to flip the output of the teacher model back to the original input to enforce consistency correctly. A combined loss function $\mathcal{L}_{comb}$ is used to update the student's weights. This loss adds the segmentation loss $\mathcal{L}_{seg}$ to the consistency loss $\mathcal{L}_{con}$ controlled by a consistency weight hyperparameter λ_{con}.

$$\mathcal{L}_{con} = \|m_s(x) - \phi(m_t(\phi^{-1}(x))\|^2 \quad (2)$$

$$\mathcal{L}_{comb} = \mathcal{L}_{seg} + \lambda_{con}\mathcal{L}_{con} \quad (3)$$

We use the combined loss shown in Equation 3 to update the weights of the mtU-Net student model. The teacher's weights are updated through an EMA of the student's weights, controlled by a decay rate hyperparameter we choose as $\rho = 0.99$. The consistency weight is controlled by a sigmoid ramp-up function with a length of 300 epochs and a maximum value $\lambda_{con} = 0.1$.

1.3. Implementation details

All models are trained with a 3D architecture, for a total of 1000 epochs, with a batch size of 3, 250 batches per epoch, and patch size of 320x320x16, using 5-fold cross validation and early stopping based on internal cross-validation metrics. The initial learning rate is 0.01, with a scheduler reducing the learning rate based on validation performance. We employed an SGD optimizer with Nesterov momentum (0.99) and weight decay of 3×10^{-5}. For the baseline supervised and semi-supervised methods, we use the publicly available weights of the PI-CAI challenge baselines repositories. The computational environment consisted of an NVIDIA GeForce RTX 2080 Ti GPU, PyTorch v1.12.1, and CUDA v11.6.0.

Data augmentation was performed using the nnU-Net framework [11]. This included random (20% probability) scaling (range 0.7–1.4), rotation (axial plane ±180°, other planes ±30°), mirroring across all axes, and random (30% probability) gamma correction (range 0.7–1.5). Spatial transformations were applied primarily within the high-resolution plane. For mtU-Net, both student and teacher models process inputs with the same augmentation policy. To enforce consistency regularization, the teacher's input undergoes an additional, reversible horizontal flip. Its output is then inversely transformed to match the student's orientation to compute the consistency loss.

Prior to model evaluation, ADC and high-b-value DWI sequences were resampled to match the T2W geometry using linear interpolation. The expert annotations were resampled to the same space using nearest-neighbor interpolation to preserve the discrete label boundaries. All MRI scans were pre-processed using the standard nnU-

Net preprocessing pipeline [11]. This process includes reorienting each volume to a consistent anatomical alignment and cropping it to the minimal non-zero bounding box. Based on the dataset's median voxel spacing, all three input sequences were resampled to a uniform resolution of 0.5x0.5x3.0 mm, assuming registered sequences. Each imaging modality was normalized by clipping voxel values to a channel-specific percentile range, followed by z-score standardization. No organ-specific mask was used for normalization. To account for the larger field of view in the PROMIS dataset, the images were cropped around the prostate gland with a 5 cm margin. The prostate mask was obtained by applying a nnU-Net segmentation model trained on the ProstateX dataset, and checked by the radiologist during the lesion annotation process.

1.4. Annotation comparison

To visualize the typical locations of lesions identified by each annotation source (AI-generated, human-annotated, and pathology-confirmed from PROMIS), binary lesion masks were aggregated across all patients within each respective group. These aggregate 3D distributions were then projected onto a 2D plane by summing voxel presences along the z-axis. Relative coverage was computed based on the 3D volume covered by the AI annotations and the volume covered by the human annotations. These volumes were then expressed as percentages relative to the total 3D volume covered by the union of both masks. The 3D Intersection over Union (IoU) was calculated between the complete AI-generated segmentation mask and the complete human-annotated segmentation mask for each of the 205 patients. The mean of these 205 patient-level IoU values was reported to summarize overall spatial agreement per case. To assess the degree to which AI annotations localized individual human-annotated lesions (used as the reference standard for this specific comparison), a lesion-level hit ratio was calculated. A 'hit' was defined as any individual human-annotated lesion achieving a 3D IoU of 0.10 or greater with any overlapping region from the corresponding AI-generated mask. The hit ratio was calculated as the total number of 'hit' human lesions divided by the total number of human-annotated lesions.

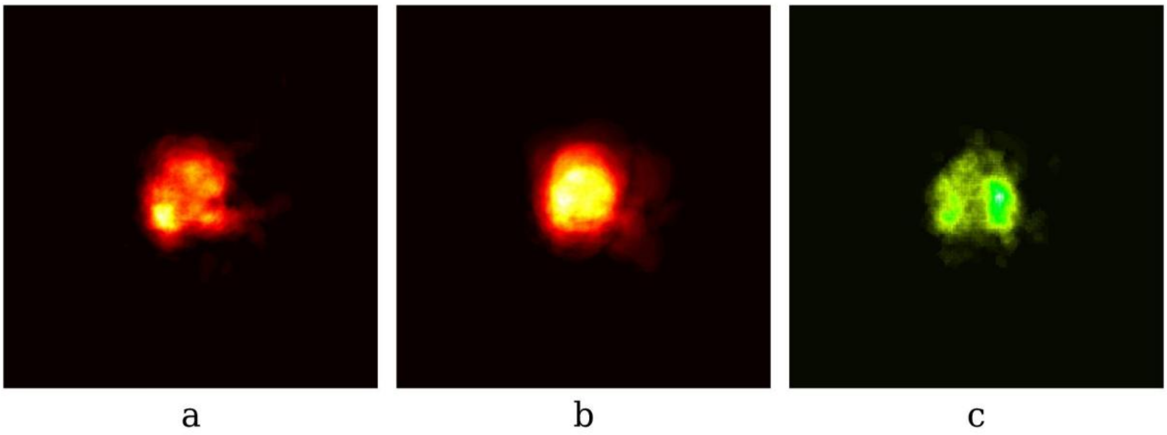

Figure 1: Location heatmap of (a) AI-annotated lesions, (b) human-annotated lesions, and (c) histology-aware annotations from the external validation (PROMIS). Computed on all slices by flattening the Z axis.

A comparison was performed between the human-annotated and AI-generated lesions within the 205 PI-CAI training samples for which both annotation types were available. Heatmaps illustrate distinct spatial distributions for AI-generated lesions (Figure1a) compared to human-delineated lesions (Figure 1b). Quantitative analysis revealed differences in spatial coverage and overlap between the annotation types. Human annotations encompassed 83.5% of the total lesion area, whereas AI annotations covered 39.1%. The mean IoU between AI and human annotations was 22.6%. Applying a hit criterion of IoU>0.10 to assess overlap, the hit ratio between the two annotation sets was 59.9%. Furthermore, the spatial distribution of pathology-aware annotations in the external PROMIS dataset is shown in Figure 1c.

We observed differences between the human and AI annotations, specifically the larger surface area and broader distribution of human-annotated lesions versus the localized and conservative nature of AI annotations. This suggests that AI models, when trained mostly on AI-annotated data, may prioritize precision over recall, potentially reducing sensitivity. The conservative annotation style on the AI-annotated data, focusing on smaller areas with a higher probability of aggressive cancer, indicates a need for careful consideration when using such data for training, particularly in clinical applications where sensitivity is crucial. Furthermore, the distinct lesion distribution observed in the PROMIS samples underscores that AI models are subject not only to technical variability in bpMRI acquisition but also to variability between populations, and ground-truth annotation. This highlights the importance of validating models on diverse external datasets to ensure generalizability.
